# Recent advances in the diagnosis of intestinal tuberculosis

**DOI:** 10.1186/s12876-022-02171-7

**Published:** 2022-03-01

**Authors:** Hasan Maulahela, Marcellus Simadibrata, Erni Juwita Nelwan, Nur Rahadiani, Editha Renesteen, S. W. T. Suwarti, Yunita Windi Anggraini

**Affiliations:** 1 Division of Gastroenterology, Internal Medicine Department, Faculty of Medicine Universitas Indonesia-Cipto Mangunkusumo National Central General Hospital, Infectious Diseases and Immunology Research Center, Indonesian Medical Education and Research Institute (IMERI), Jakarta, Indonesia; 2 Division of Tropical Medicine and Infectious Diseases, Internal Medicine Department, Faculty of Medicine Universitas Indonesia, Cipto Mangunkusumo Hospital, Infectious Diseases and Immunology Research Center, Indonesian Medical Education and Research Institute (IMERI), Jakarta, Indonesia; 3 Department of Anatomical Pathology, Faculty of Medicine Universitas Indonesia-Cipto Mangunkusumo National Central General Hospital, Jakarta, Indonesia; 4 Infectious Diseases and Immunology Research Center, Faculty of Medicine Universitas Indonesia, Indonesian Medical Education and Research Institute (IMERI), Jakarta, Indonesia

**Keywords:** Intestinal tuberculosis, Diagnosis, GenExpert, IGRA, PCR, Multiplex-PCR, Immunological marker

## Abstract

**Background:**

Intestinal tuberculosis still has a high incidence, especially in developing countries. The biggest challenge of this disease is the establishment of the diagnosis because the clinical features are not typical. Investigations such as culture, acid-fast bacilli (AFB) staining, and histopathology have low sensitivity, so other investigations are needed. Latest molecular-based diagnostic modalities such as GeneXpert, interferon-gamma (IFN-γ) release assays (IGRA), polymerase chain reaction (PCR), multiplex-PCR, and immunological markers are expected to help diagnose intestinal tuberculosis. This article review will examine the latest diagnostic modalities that can be used as a tool in establishing the diagnosis of intestinal tuberculosis.

**Results:**

Through a literature search, we were able to review the diagnostic values of various available diagnostic modalities as the appropriate additional test in intestinal tuberculosis. Culture as a gold standard has a sensitivity and specificity value of 9.3% and 100% with the MGIT BACTEC system as the most recommended medium. The sensitivity values of AFB staining, histopathology examination, GeneXpert, IGRA, PCR, multiplex-PCR and, immunological markers were ranged between 17.3 and 31%; 68%; 81–95.7%; 74–88%; 21.6–65%; 75.7–93.1%; and 52–87%, respectively. Meanwhile the specificity values were 100%; 77.1%; 91–100%; 74–87%; 93–100%; 96.4–100%; and 70–95%, respectively.

**Conclusion:**

The combination of clinical examination, conventional examination, and the latest molecular-based examination is the best choice for establishing the diagnosis of intestinal tuberculosis. Most recent modalities such as multiplex PCR and immunological marker examinations are diagnostic tools that deserve to be used in diagnosing intestinal tuberculosis as their sensitivity and specificity values are quite high and more evidences are expected to support the application of these examinations shortly soon.

## Introduction

Tuberculosis is an infectious disease caused by *Mycobacterium tuberculosis*. These bacteria generally infect the lung tissue, but can also infect outside of the lung. This condition is called extrapulmonary tuberculosis. Tuberculosis is 13th leading cause of death worldwide and also it is known that tuberculosis is the second leading infectious killer after COVID-19. The prevalence of this disease is still quite high, especially in developing countries. In 2020, 10 million cases of tuberculosis were found worldwide with 1.5 million people died from tuberculosis [1]. It is estimated that a quarter of the world's population has latent tuberculosis. Extrapulmonary tuberculosis occurs in 20% of tuberculosis patients. Meanwhile, 10% of all cases of extrapulmonary tuberculosis are intestinal tuberculosis. Intestinal tuberculosis has a poor prognosis, especially if there are life-threatening complications such as intestinal stricture, obstruction, perforation, and bleeding [2–4].

The method of establishing a diagnosis is the biggest problem in the management of patients with intestinal tuberculosis. This is caused by imprecise clinical manifestations and can resemble a variety of other diseases. That is the reason why intestinal tuberculosis is called *the great mimicker*. Gold standard examination for intestinal tuberculosis is a culture of *M. tuberculosis* using intestinal mucosal tissue specimens. However, the paucibacillary nature of these bacteria makes it difficult to detect *M. tuberculosis* using this method, so the risk of false negatives is very likely. Therefore, various types of methods for establishing the diagnosis of intestinal tuberculosis have been developed by combining clinical examination with other conventional examinations such as colonoscopy histopathology examination of intestinal mucosal tissue, acid-fast bacilli (AFB) staining, culture using a variety of the latest medium, and radiological examination such as computed tomography (CT) scan or magnetic resonance imaging (MRI). The use of various diagnostic modalities still has many shortcomings because several other diseases also have findings that are very similar to intestinal tuberculosis, for example; Crohn’s disease and intestinal carcinoma. Difficulties in establishing a diagnosis of intestinal tuberculosis can cause under- or over-diagnosis which results in errors in the administration of therapy [2, 4, 5].

At this present time, various types of new diagnostic methods have been developed that can help the process of diagnosing tuberculosis, including molecular-based examinations such as GeneXpert, polymerase chain reaction (PCR), multiplex-PCR, and immunological markers examination. All these tests have their advantages and disadvantages, so a critical review of the diagnostic modality is needed before it is applied to patients suspected of intestinal tuberculosis [4, 6].

This article review will examine the latest diagnostic modalities that can be used as a tool in establishing the diagnosis of intestinal tuberculosis. We hoped that the article can assist health care providers in deciding which diagnostic modalities can be used in patients with suspected intestinal tuberculosis to prevent under- or over-diagnosis.

## Clinical manifestations of intestinal tuberculosis

The clinical features of intestinal tuberculosis vary greatly and are not specific. This makes it difficult to distinguish intestinal tuberculosis from other intestinal diseases. A Comparison of the clinical finding according to pieces of literature is summarized in Table 1.

**Table 1:** Clinical manifestations of intestinal tuberculosis

Clinical manifestation	Shi et al. [5] N (%)	Patel et al. [8] N (%)	Gan et al. [7] N (%)	Tanoglu et al. [36] N (%)	Cheng et al. [37] N (%)
	85 pt (China)	69 pt (India)	81 pt (China)	104 pt (Multicenter)	85 pt (China)
Abdominal pain	70 (82.4)	53 (76)	87 (87.7)	80 (76,9)	75 (88,2)
Weight loss	62 (72.9)	42 (60.87)	65 (80.2)	52 (50)	64 (75,3)
Fever	55 (64.7)	50 (72.46)	35 (43.2)	69 (66,3)	44 (51,8)
Anemia	55 (64,7)	Unknown*	52 (64.2)	94 (90,4)	50 (58,8)
Poor appetite	48 (56.5)	Unknown*	Unknown*	94 (90,2)	Unknown*
Diarrhea	43 (50.6)	20 (28.99)	38 (46.9)	25 (24)	30 (35,3)
Night sweat	25 (29.4)	Unknown*	25 (30.9)	70 (67,3)	32 (37,6)
Nausea and vomiting	Unknown*	Unknown*	Unknown*	46 (44,2)	31 (36,5)
Abdominal mass	9 (10.6)	7 (10.15)	5 (6.2)	See in the text	15 (17,6)
Ascites	9 (10.6)	7 (10.15)	28 (34.6)	26 (25)	20 (23,5)
Alternating diarrhea and constipation	6 (7.1)	Unknown*	31 (38.3)	Unknown*	2 (2,4)
Constipation	3 (3.5)	5 (7.25)	13 (16.0)	22 (21,2)	6 (7,1)
Partial intestinal obstruction	16 (18.8)	7 (10.15)	Unknown*	Unknown*	43 (50,6)
Intestinal bleeding	9 (10.6)	10 (14.5)	8 (9.9)	11 (10,6)	Unknown*
Bowel fistula	3 (3.5)	Unknown*	Unknown*	Unknown*	1 (1,2)
Bowel perforation	1 (1.2)	Unknown*	Unknown*	Unknown*	21 (24,7)
Extraintestinal manifestation	Unknown*	10 (14.5)	Unknown*	Unknown*	Unknown*

^*^Not mention in the study

The most common clinical features are abdominal pain, weight loss, and fever. Abdominal pain is chronic but can also be acute on chronic if acute complications occur. Abdominal pain often occurs in the right lower quadrant area of the abdomen and the periumbilical area. Weight loss is also the most common symptom that occurs in patients with intestinal tuberculosis due to various causes such as chronic inflammatory processes, decreased intake, and impaired absorption. Weight loss can be accompanied by mild to moderate anemia. Most intestinal tuberculosis patients also experience irregular low-grade fever, with body temperature between 37.5 and 38.5 °C, accompanied by night sweats. An increase in temperature occurs more frequently in the afternoon. Other gastrointestinal symptoms also often occur such as chronic diarrhea, constipation, and decreased appetite. On physical examination, it is often found ascites and palpable abdominal mass, especially in the right lower quadrant area (19,3%), and splenomegaly (14,2%). Complications that are often found are intestinal bleeding, fistula, and perforation [5–8].

Imaging of intestinal tuberculosis can also be done to support diagnosis of intestinal tuberculosis. In duodenal tuberculosis, we can find compression of adjacent lymphadenopathy that can obstruct the lumen and thickening of duodenal walls on CT scan [9]. In ileocaecal tuberculosis which is very common (80–90%) [10], we can find mild to eccentric mural thickening of the ileocaecal and it can also involve the medial caecal wall and valve. These signs can easily be found on CT scan. Meanwhile, in colonic tuberculosis, strictures, features of colitis, and polypoid lesions are the most common finding on CT scan [11]. Ultrasonography and CT scan may also show generalized or local ascites of abdomen. However, these radiological findings are not specific [12].

Moreover, patients with intestinal tuberculosis can also be presented as asymptomatic. One study in Japan published asymptomatic patients with confirmed intestinal tuberculosis. Among 11 patients who were confirmed with intestinal tuberculosis, only one patient showed symptoms of weight loss and anorexia. There were case reports that report findings of inadvertently increased 18F-fluorodeoxyglucose activity in the ileocecal area in patients undergoing positron emission tomography (PET) to rule out lung carcinoma. Then ileocecal resection surgery is performed and it was confirmed as intestinal tuberculosis. The patient had no gastrointestinal symptoms at all. One literature also mentions that as many as 53% of patients with intestinal tuberculosis were accidentally detected when a surgical procedure was performed with an incorrect diagnosis. Due to the non-specific nature of intestinal tuberculosis symptoms, various diagnostic modalities have been under study and research over the following decades. A combination of history taking, physical examination, and several diagnostic modalities is needed to promptly diagnose the disease [8].

## Diagnostic modalities of intestinal tuberculosis

### Microbiological examination

**Fig. 1:**
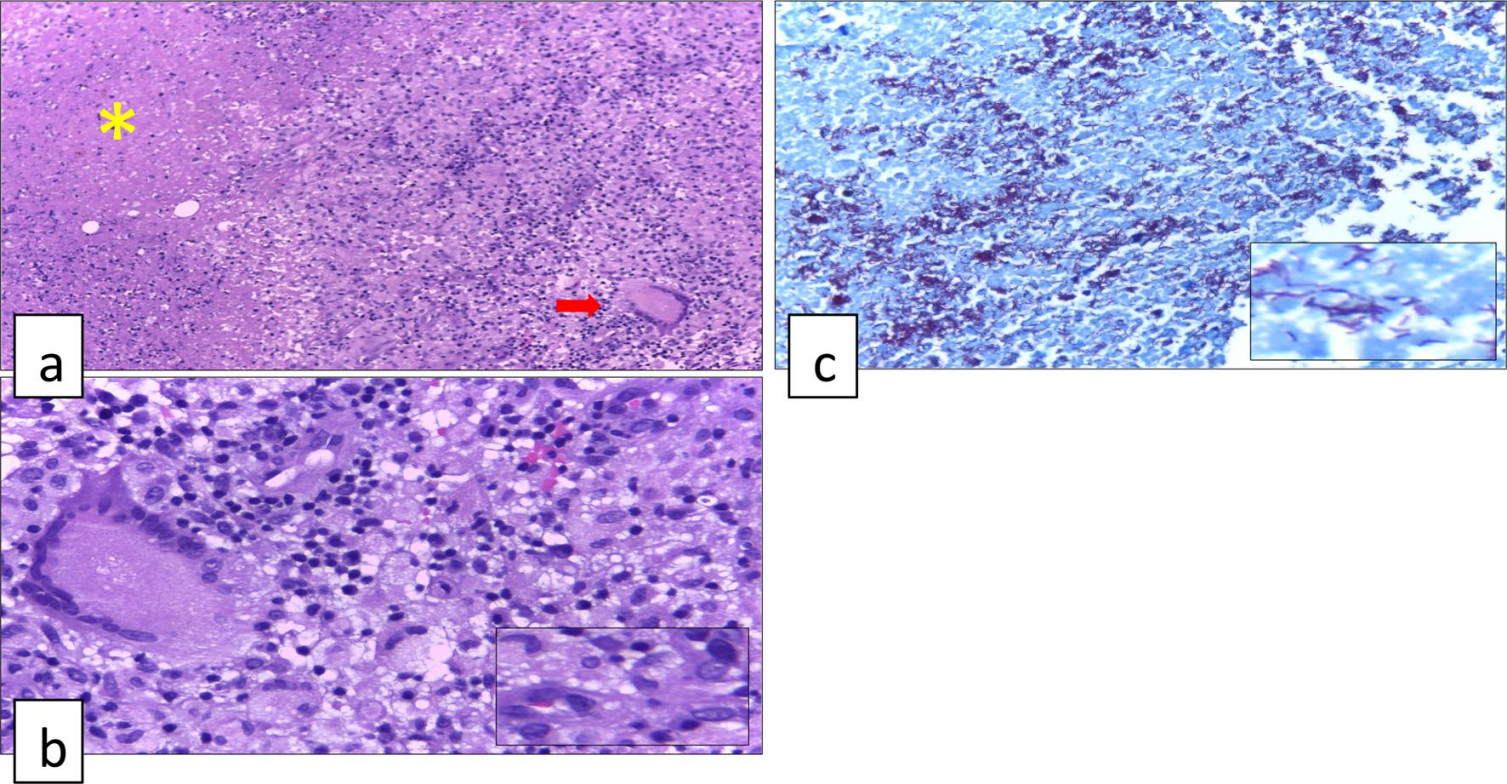
Histopathology features often appear in intestinal tuberculosis **a** granuloma consisted of lymphocytes, histiocytes, Langerhans giant cell (red row) and caseating necrosis (yellow Asterix) (4 × , Hematoxylin–Eosin staining), **b** cluster of histiocytes (inset) with one Langerhans cell containing numerous nuclei (40 × , Hematoxylin–Eosin staining), **c** Ziehl-Nielsen staining showing acid-fast bacilli in red color

One microbiological examination to diagnose intestinal tuberculosis is acid-fast bacilli (AFB) staining. An example of the acid-fast bacilli (AFB) staining methods that are often used is the *Ziehl Nelsen* (ZN) method. ZN is a conventional examination to identify *M. tuberculosis* and *M. leprae* bacteria microscopically. This examination utilizes the presence of mycolic acid in the cell wall of mycobacteria which can hold the carbol fuschin solution (colored red) inside the cell wall even though it has been given an acid solution. Positive results will give a picture of the appearance of reddish-colored bacilli bacteria with a blue background (Fig. 1). The best specimen for this examination is ileocecal mucosal biopsy tissue. This examination has very high specificity of 100% so that patients with positive smear can be confirmed as intestinal tuberculosis. However, the disadvantage of this examination is its low sensitivity, so the risk of experiencing false negative is very high. The sensitivity of ZN staining is around 17.3–31% and it will increase with the number of specimens examined. Despite having a low sensitivity value, this examination is still highly recommended to be performed routinely in patients with intestinal tuberculosis as an indicator in assessing treatment response [4, 8].

Another microbiological examination is the *M. tuberculosis* culture. This examination is a gold standard for diagnosing intestinal tuberculosis. The specimen used is a biopsy tissue on the ileal mucosa, caecum, and colon. In general, there are three types of medium for *M. tuberculosis* culture; egg-based (*Lowenstein-Jensen* / LJ), agar-based (Middlebrook 7H11), and liquid-based (Middlebrook 7H12) medium. However, currently, the latest medium for *M. tuberculosis* that has been widely used and accepted is the radiometric BACTEC 460 system, the Microbacteria Growth Indicator Tube (MGIT) BACTEC 960 system, and the EPS II system. The BACTEC system was developed by *Becton Dickson* using radioactive carbon dioxide from a palmitic acid substrate. MGIT system uses a non-radioactive basis namely fluorochromes to detect bacterial growth and drug resistance. The use of this medium is very helpful in detecting the presence of bacteria in the initial phase of infection (7–12 days). At this present time, the most recommended culture medium is MGIT medium, because this medium has several advantages such as not containing radioactive substances, does not require CO_2_ tanks, and minimal risk of contamination. After all, monitoring of this culture can be done non-invasively and the use of screwcaps on tubes eliminates the need for use of needles to reduce the risk of needling. Studies showed that MGIT BACTEC has the quickest recovery time and the highest positive sample rate compared to LJ and Middlebrook 7H10 medium [5]. However, it is important to note that the difference in sensitivity between MGIT BACTEC 960 and LJ medium does not differ greatly, so LJ medium is still recommended, especially in areas with limited diagnostic facilities [8, 13].

*M. tuberculosis* culture is a very specific examination, where the specificity value reaches 100%, but the sensitivity value is only 9.3% with a positive predictive value (PPV) of 100% and a negative predictive value (NPV) of 38.29%. As a gold standard examination, culture examination is still recommended especially for patients who will undergo a colonoscopy procedure and take a tissue specimen [6].

### Histopathological examination

One of the conventional tests used to diagnose intestinal tuberculosis is histopathological examination. Specimens are obtained from a biopsy in a colonoscopic or laparoscopic procedure. Some typical histopathological features that often appear in mucosal tissue with intestinal tuberculosis; granuloma with caseating necrosis, Langerhans giant cell, conglomerate epithelioid histiocytes, and disproportionate submucosal inflammation. The table below shows five pieces of literature that compare the most common histopathological findings in confirmed cases of intestinal tuberculosis (Table 2). It is widely known that former literature described histopathological findings in intestinal tuberculosis as typical with granuloma and caseating necrosis. Oddly enough, a study reported that only 13–33% of patients with intestinal tuberculosis showed those findings. As a result, the sensitivity of this examination is quite low at around 68%, with a specificity of 77.1%, PPV 68%, and NPV 77.1% [6].

**Table 2:** Histopathological feature of intestinal tuberculosis [36–40]

	Method	Location	N	Granuloma (%)	Caseating Necrosis (%)	Langhans (%)	Giant Cell (%)	Ulcer (%)
Tanoglu et al. [36]	Retrospective	Multicenter*	82	36,6	63,4	N/A	N/A	N/A
Cheng et al. [37]	Retrospective	China	85	70,6	24,7	N/A	N/A	65,9
Bandi et al. [38]	Cross sectional	India	135	97,3	79,26	95,56	15,56	N/A
Limsrivilai et al. [39]	Retrospective	Thailand	83	71,1	N/A	N/A	N/A	44,6
Jin et al. [40]	Retrospective	South Korea	52	46,2–57,7**	26,9	N/A	45,5	30,9

^*^Multicenter studies in UK, France, Belgium, Italy, Turkey, Kazakhstan, Saudia Arabia, and Egypt

^**^Confluent granuloma up to 46,2% and discrete granuloma up to 57,7%

The number of biopsy specimens will greatly influence the results of histopathological examination. The specimen volume, depth of specimen collection, and location of specimen collection result in the better diagnostic accuracy of this examination. Some literature suggests taking at least 8–10 biopsy specimens. Taking a minimum of eight biopsy samples has a higher diagnostic accuracy of 11.4%, compared to taking four biopsy samples [6, 8, 13].

### Combination of histopathological examination and M. tuberculosis culture

Histopathological examination and culture of *M. tuberculosis* have their respective advantages and disadvantages, so it is advisable to do a combination of the results of both tests. The combination of these two will increase diagnostic accuracy by 17%. Several studies reported a comparison of combined histopathological examination with M Tb culture in regards to culture medium; LJ and BACTEC medium (Table 3). It can be concluded that BACTEC medium has better diagnostic value compared to LJ medium in a scenario of combined histopathological examination and culture [6].

**Table 3:** Diagnostic yield of combination between histology and culture [6]

Study	Patients with ileocolonic TB (N)	Histology N (%)	Culture positivity N (%)	Culture medium	Combined diagnostic yield%
Vij et al	28	21 (75)	13 (46)	LJ medium	75
Amarapurkar et al	26	13 (50)	6 (23)	BACTEC	Not commented
Shah et al	50	40 (80)	3 (6)	LJ medium	80
Leung et al	23	3 (13)	17 (73)	BACTEC	82
Krisch et al	18	14 (78)	14 (78)	BACTEC	78
Samant et al	61	48 (78.6)	31 (50.8)	BACTEC	91.8

### GeneXpert

GeneXpert assay is a fully automated real-time PCR-based test designed for rapid and simultaneous detection of mutations related to the resistance of *M. tuberculosis* to rifampicin. GeneXpert is considered very effective because it has high sensitivity and specificity and the results of the examination can be obtained in a short time (about 2 h). This examination has been routinely carried out to establish diagnosis of pulmonary tuberculosis, where sensitivity can nearly reach 100% by using specimens such as sputum or bronchoalveolar lavage [6, 14]. A new generation of Xpert has been developed termed Xpert MTB/RIF Ultra assay (Ultra). A study conducted by Chakravorty et al. found that the sputum sensitivity of Ultra versus Xpert is 87.5% versus 81%, respectively. Meanwhile both specificity is 98.7% [15].

Some studies also apply this examination to establish a diagnosis of extrapulmonary tuberculosis including intestinal tuberculosis (Table 4). The sensitivity and specificity of this examination are quite high, so this examination is quite recommended to be performed in patients with suspected intestinal tuberculosis. However, the disadvantage of this examination is that it is not available in many hospitals. Despite having good diagnostic accuracy, this examination still cannot replace microbiological examination as a gold standard examination [6, 14].

**Table 4:** GeneXpert in intestinal tuberculosis [14]

Study	GeneXpert	
	Sensitivity (%)	Specificity (%)
Singh et al	88	91
Kumar et al	81	100
Lowbridge et al	95.7	100

The study conducted by Talib et al. provides a new perspective in carrying out the GeneXpert examination. Talib et al. used specimens in the form of feces, while previous studies used biopsy specimens from ileocecal tissue. The results of the study stated that the sensitivity of this examination was 39.1% and specificity 85.7%. Based on this result, it can be concluded that fecal samples can increase the risk of false negatives greater compared to biopsy tissue specimens. Therefore, the use of biopsy tissue samples is recommended for GeneXpert examination in cases of intestinal tuberculosis [6, 14].

### Interferon-gamma release assay (IGRA)

Currently, T-cell based interferon-gamma (IFN-γ) release assays (IGRA) have progressively been used to subtitute tuberculin skin test (TST) as an instrument to diagnose tuberculosis. IGRA has been proved to have higher accuracity. With TST, patients with Bacillus Calmette-Guérin (BCG) vaccination have a great number of false-positive results and also, in immunosuppressed patients, it shows false-negative results. *M. tuberculosis* specific region of difference 1 (RD1) antigens like culture filtrate protein 10 (CFP-10) and early secretory antigen target 6 (ESAT-6) were found as a consequence of developments in microbiology. Absent in BCG and most environmental mycobacteria, with the exception of *M. kansasii*, *M. szulgai*, and *M. marinum*, these antigens form the basis of IGRA, which shows the presence of tuberculosis infection by detecting the in vitro release of interferon-gamma upon stimulation from the sensitized T cells [16, 17]. IGRA has two internationally commercial techniques: QuantiFERON®-TB Gold In-Tube (QFT-GIT) (Cellestis, Carnegie, Australia) and T-SPOT®.TB (Oxford Immunotec, Abingdon, UK). The QFT-GIT read the concentration of interferon gamma (IFN- γ) via an enzyme-linked immunosorbent assay, meanwhile T-SPOT read the number of IFN-gesecreting T cells via an enzyme-linked immunospot assay. Both tools are approved by the FDA (US Food and Drug Administration) and EC (European Committee) [18].

Assay manufacture, pre-analytical processing, analytical factors, and immunomodulation were mentioned can affect the IGRA value. Numerous risk factors were also mentioned to be related to negative results of IGRA such as immunodeficiency, young or advance age, TST, extra pulmonary tuberculosis, disseminated tuberculosis, concomitant tuberculosis, and smoking. The immune-mediated inflammatory diseases (IMID) type may also affect the clinical accuracy of IGRA differentially to diagnose latent tuberculosis. Crohn’s disease may negatively affect the accuracy of IGRA when compared to psoriasis or inflammatory rheumatic diseases [17].

A study conducted by Zhao et al., with 56 patients suspected Crohn’s disease enrolled, concluded that patients with IGRA ⩾100 pg/ml were indicated to a high possibility of tuberculosis infection with sensitivity and specificity value 88% and 74%, respectively. In this study they got 33 patients with IGRA ⩾100 pg/ml. Twenty five patients with IGRA ≥ 100 pg/ml received anti tuberculosis therapy and they had been reported to have clinical improvement after around 2–3 months after admission of anti tuberculosis therapy. Thus, they drew a conclusion that IGRA ≥ 100 pg/ml is associated with higher probability of tuberculosis infection over Crohn’s disease. Meanwhile in intestinal tuberculosis patients, 8 from15 patients with IGRA ≥ 400 pg/ml had weight loss symptom, while only 1 from 18 patients with IGRA < 400 pg/ml had weight loss symptom. From these data, Zhao et al. also concluded that IGRA value may also correlate positively with the severity of intestinal tuberculosis. However, because the sampel is too small, conclusions tht we mentioned above are still need to be reassured in future studies [19].

Some studies have been done to show the sensitivity and specificity of IGRA for diagnosing intestinal tuberculosis. In this study, we included 4 literatures of intestinal tuberculosis (Table 5) which discussed about the effectiveness of IGRA test using blood sample. The average sensitivity of these study range from 74 to 88%, and the specificity range from 74 to 87% [18–21].

**Table 5:** Diagnostic accuracy of IGRA [18–21]

Study	Sample	Techniques	IGRA	
			Sensitivity (%)	Specificity (%)
Ng et al. [18]	Blood	T-SPOT.TB or QFT-GIT	81	85
Zhao et al. [19]	Blood	**-**	88	74
Chen et al. [20]	Blood	T-SPOT.TB or QFT-GIT	74	87
Limsrivilai et al. [21]	Blood	**-**	84	86

### Polymerase chain reaction (PCR)

Polymerase chain reaction (PCR) is a common laboratory technique used to make many copies of a particular region of deoxyribonucleic acid (DNA). A basic principle of PCR is using the ability of DNA polymerase to synthesize the new strand of DNA complementary to the predefined template strand. Therefore, this examination can be used to detect the presence of *M. tuberculosis* bacteria. The PCR procedure can be divided into three parts: DNA extraction, DNA amplification, and DNA detection. The target sequence for tuberculosis PCR amplification is the IS6110 gene. This gene is a specific gene segment of *M. tuberculosis* bacteria that is not found in other mycobacterial bacteria [22].

Several studies have tried to implement PCR as a diagnostic modality for intestinal tuberculosis. Some studies use ileocecal mucosal biopsy tissue specimens and also fecal specimens. Fei et al. compared the two types of specimens and found that fecal specimen would give a higher sensitivity value of about 30% when compared to biopsy tissue specimens. Besides, the method of collecting fecal specimen is easier compared to biopsy, so the use of fecal specimens is quite recommended especially in limited facilities [4, 6, 22].

Table 6 shows several studies assessing the effectiveness of PCR examination in cases of intestinal tuberculosis with biopsy tissue specimens. From this table, it can be concluded that the specificity of PCR is high enough to establish a diagnosis of intestinal tuberculosis, but this examination has low sensitivity, so patients with negative PCR results still have the possibility of intestinal tuberculosis (high false negative). Some factors that cause the low sensitivity of this examination are the taking of specimens that are too little or too deep which causes the amount of extracted *M. tuberculosis* DNA to be small or even undetectable. The second factor is the use of paraffin-embedded biopsy specimens that can degrade bacterial DNA so that it cannot be detected, and the last factor is PCR of *M. tuberculosis* that only detects the IS6110 gene sequence so that if the bacteria are in an imperfect condition and does not have the gene sequence, it can show negative results. Therefore, it is hoped that the PCR will be able to detect more gene sequence for tuberculosis, to increase the diagnostic value of it [4, 6, 22].

**Table 6:** PCR in intestinal tuberculosis [4, 6, 22]

Study	Polymerase chain reaction	
	Sensitivity (%)	Specificity (%)
Lowbridge et al	50	100
Mehta et al	21.6	95
Fei et al	55	94
Yuan et al	58	93
Deepak et al	65	100
Jin et al	36	100
Pulimood et al	30	95
Amarapukar et al	22	95
Gan et al	64	100

## Multiplex-polymerase chain reaction

Multiplex-polymerase chain reaction (multiplex-PCR) is a widespread molecular biology technique for amplification of multiple targets in a single PCR experiment. Using this technique, more than one target gene sequence in a clinical specimen can be amplified in a single tube. Several studies have tried to apply this examination to establish the diagnosis of intestinal tuberculosis and compare it with other conventional examinations including simple PCR examination [23, 24].

The advantage of multiplex-PCR compared to simple PCR is can detect more than one gene target, so it has a lower false-negative result. The difference between simple PCR and multiplex-PCR for the diagnosis of intestinal tuberculosis is that simple PCR only amplifies one gene sequence, namely IS6110, while multiplex-PCR can detect the IS6110 gene and other mycobacteria-specific gene sequences such as the MPB64 gene, and the 16SrRNA gene. Simple PCR has lower sensitivity because several risk factors can cause the IS6110 gene sequence to be detected. Numerous studies prove that the IS6110 gene sequence is not always found in *M. tuberculosis* bacteria, so false negativity is very likely to occur. Meanwhile, a multiplex-PCR examination has the advantage of being able to detect other specific gene sequences (more than one type of gene sequence), so the sensitivity of this examination is certainly higher when compared to simple PCR [23–25].

Several studies have assessed the effectiveness of multiplex-PCR as a modality for diagnosing pulmonary and extrapulmonary tuberculosis including intestinal tuberculosis (Table 7). Table 7 shows that the effectiveness of multiplex-PCR for cases of pulmonary tuberculosis and extrapulmonary has almost the same effectiveness. All studies state that the sensitivity and specificity of this examination is high enough that it can be recommended as a modality for establishing the diagnosis of tuberculosis including intestinal tuberculosis [26, 27].

**Table 7:** Multiplex-polymerase chain reaction [23–27]

Study	Diagnosis	Multiplex-polymerase chain reaction	
		Sensitivity (%)	Specificity (%)
Bhawsar et al	Pulmonary tuberculosis	93.1	96.5
Kulkarni et al	Pulmonary tuberculosis	81.7	97.3
Dahiya et al	Pleural tuberculosis	89.6	96.7
Hallur et al	Peritoneal tuberculosis	75.7	100
Hallur et al	Intestinal tuberculosis	87.5	96.4
Sharma et al	Intestinal tuberculosis	77.5	100
Malik et al	Intestinal tuberculosis	87.5	100

Studies by Kulkarni et al. compared simple PCR examination with multiplex-PCR as a modality for diagnosing tuberculosis. It stated that multiplex-PCR has much higher sensitivity compared to simple PCR. Multiplex PCR can detect the presence of *M. tuberculosis* even in small amounts (paucibacillary) or in conditions where the IS6110 gene sequence is not found. Therefore, it can be concluded that multiplex-PCR examination can cover the shortcomings of simple-PCR. Kulkarni et al. also compared the multiplex-PCR examination with the gold standard examination for tuberculosis. The microbiological examination carried out was AFB staining (ZL staining) and *M. Tuberculosis* culture using LJ medium. The multiplex-PCR examination has been shown to have a value of greater sensitivity than microbiological examination and its specificity is close to 100% as it is shown in Table 8. Therefore, it can be concluded that multiplex-PCR examination can be recommended as a supporting examination that can support the diagnosis of tuberculosis, both pulmonary and extrapulmonary tuberculosis such as intestinal tuberculosis [23–27].

**Table 8:** Comparison between multiplex-PCR and microbiological findings in intestinal tuberculosis [27]

Diagnostic modality	Sensitivity (%)	Specificity (%)
AFB Smear microscopy	53.3	100
LJ Culture	69.2	100
Multiplex-PCR	81.7	97.3

### Immunological markers

Intestinal tuberculosis has several differential diagnoses that are quite difficult to distinguish, especially Crohn’s disease. The two diseases have great similarities both from symptoms to appearance on colonoscopy examination. The paucibacillary nature of *M. tuberculosis* bacteria make it more difficult to detect these bacteria so that it is more difficult to distinguish between the two diseases. Some researchers have developed diagnostic modalities to distinguish between intestinal tuberculosis and Crohn's disease. Here, we would discuss diagnostic modality of immunological markers of peripheral blood and histopathological specimens [28–32].

Immunological marker of pheripheral blood that can be used is Forkhead box P3 (FOXP3). FOXP3 will express CD4^+^ CD25^+^ T-regulatory cells (Tregs), the key regulators of mucosal immune responses that play a role in chronic inflammation and infectious diseases. In general, FOXP3 Treg of peripheral blood is lower in patients with Crohn's disease patients, but higher when it was performed on the intestinal biopsy specimens. During active Crohn's disease, these regulatory cells are sequestered to the sites of active inflammation which are in the intestine, so the levels in the peripheral blood decrease. On the contrary, infectious diseases such as tuberculosis have consistently demonstrated higher frequencies of FOXP3 in peripheral blood. The theory supports the study conducted by Rampal et al. on the role of FOXP3 T-regulatory cells as an immunological marker to distinguish between intestinal tuberculosis and Crohn's disease. The study used specimens from peripheral blood to assess the levels of FOXP3 Treg cells as the main indicator. The results of the study stated that the level of FOXP3 Treg cell was significantly higher in the group of patients with intestinal tuberculosis when compared with Crohn's disease. Several previous studies stated several cutoff values of FOXP3 Treg cell levels which were considered significant to distinguish intestinal tuberculosis and Crohn's disease (Table 9). The cutoff with the best diagnostic accuracy is 32.37% [28, 33, 34].

**Table 9:** Comparison of diagnostic accuracy of FOXP3 + Treg cell enumeration [28]

	Cutoff > 32.50%	Cutoff > 31.35%	Cutoff > 32.37%
Sensitivity (%)	79	83	87
Specificity (%)	91	83	95
PPV (%)	80	91	95
NPV (%)	89	68	75

Another immunological marker that can be used is CD73 from histopatological specimens. Banarjee et al. demonstrated that granulomas of intestinal tuberculosis and Crohn’s disease can be differentiated by CD73, a mesenchymal stem cells (MSCs) surface marker expression [31]. MSCs are multi-potent stromal cells which can differentiate into a number of cell types, such as osteoblasts, chondrocytes, and adipocytes [31]. MSCs express numerous of non-specific markers, one of them is CD73. MSCs have been proved to provide a protective niche where MTB can persist, evading host immune responses and drug treatments [35]. Watermeyer et al. continued Banarjee et al. to see if CD73 is of value in differentiating intestinal tuberculosis and Crohn’s disease in a South Africa cohort. They discovered that more patients with intestinal tuberculosis express CD73 in their granolomas than those with Crohn’s disease with sensitivity value 52% and specificity value 70% [32].

These immunological markers examination can be an alternative to help establish the diagnosis of intestinal tuberculosis. However, the number of studies that examine this examination is quite minimal, thus more evidence is needed.

## Shortcoming of current diagnostic tools and future direction

*M. tuberculosis* culture and AFB staining are diagnostic tools which have very high specificity value (reach 100% specificity) but low sensitivity value (17.3–31% and 9.3%) [4, 6, 8]. The low sensitivity of these tools happened because of some possible explanations like inappropriate sample collection, receiving antimicrobials before sampling, using non-selective media from colonized sites in culture, and also inappropriate staining and assessing with microscope in AFB. In histopathological examination, the highlight problem for its diagnostic value is the biopsy sampling. As explained above, the number of biopsy specimens will greatly influence the results of histopathological examination.

IGRA, as previously explained, has numerous risk factors that associated with negative results of IGRA such as immunodeficiency, young or advanced age, TST, extra pulmonary tuberculosis, disseminated tuberculosis, concomitant tuberculosis, and smoking. The immune-mediated inflammatory diseases (IMID) type may also affect the clinical accuracy of IGRA differentially to diagnose latent tuberculosis [17]. GeneXpert, PCR, multiplex-PCR, and immunological markers have quite high sensitivity and specificity values, possibly due to their origin from molecular-based methods. These recent tools are developed in order to cover the shortcoming of the conventional tools.

From this review, we hope that clinicians could take these modalities that we explained above into a consideration in diagnosing intestinal tuberculosis in healthcare facilities. Although the availability of these tools are not evenly distributed, some diagnostic tools are worth looking for to diagnose tuberculosis, especially intestinal tuberculosis which has no specific symptoms or even asymptomatic. Further diagnostic technologies like other immunological markers from tuberculosis immune response are expected to be discovered seeing the complexities of human immunology, in order to help clinicians better in diagnosing intestinal tuberculosis.

## Conclusion

Intestinal tuberculosis is a disease called the great mimicker because it has clinical symptoms that can mimic a variety of diseases. Various modalities have been developed to find the best way to diagnose this disease. Conventional examinations such as *M. tuberculosis* culture, AFB staining, and histopathology examination have high specificity but low sensitivity. Therefore, various new molecular-based methods have been developed such as GeneXpert, IGRA, PCR, multiplex-PCR, and immunological markers.

The combination of history taking, physical examination, conventional examination, and the latest molecular-based examinations can improve the accuracy of diagnosis and prevent the under-diagnosis of intestinal tuberculosis. Most recent modalities such as multiplex PCR and immunological marker examinations are diagnostic tools that deserve to be used in diagnosing intestinal tuberculosis as their sensitivity and specificity values are quite high. More evidences are expected to support the application of these recent examinations shortly soon.

## Data Availability

Not applicable in our study.
